# Application of exogenous auxin and gibberellin regulates the bolting of lettuce (*Lactuca sativa* L.)

**DOI:** 10.1515/biol-2022-0043

**Published:** 2022-04-29

**Authors:** Yubo Wang, Bingyan Li, Yunfeng Li, Wei Du, Yueting Zhang, Yingyan Han, Chaojie Liu, Shuangxi Fan, Jinghong Hao

**Affiliations:** Beijing Key Laboratory of New Technology in Agricultural Application, Beijing University of Agriculture, National Demonstration Center for Experimental Plant Production Education, Plant Science and Technology College, Beijing University of Agriculture, Beijing, P.R. China

**Keywords:** lettuce, gibberellin, auxin, bolting, gene expression

## Abstract

Plant bolting is regulated and controlled by various internal and external factors. We aimed to provide an improved method for breeding to determine whether there is a synergism between hormones and to explore the regulatory effect of plant hormones on the bolting of leaf lettuce. Lettuce plants were sprayed with exogenous auxin and gibberellin separately or in combination. The specific bolting period was determined by the change in stem length and cytological observation. The dynamic changes in endogenous hormones and genes closely related to bolting were analyzed. Treatment with gibberellin alone and the combined application of auxin and gibberellin induced bolting on the fourth day, and treatment with auxin alone resulted in bolting on the eighth day. In the early bolting stage, the auxin contents in the stems of the treatment groups, especially the combined gibberellin and auxin group, were higher than those of the control group. After the application of exogenous auxin and gibberellin, we found that the expression of the *ARF8* and *GID1* genes was upregulated. Based on the results of our study, combined treatment with exogenous gibberellin and auxin was the best method to promote the bolting of leaf lettuce, and the *ARF8* and *GID1* genes are closely related to this process.

In lettuce (*Lactuca sativa*), the transition from the vegetative stage to the reproductive stage occurs in the meristem in the wreath. During the transition period, the apical meristem cap of vegetative branches is elongated, and then, a microflower primordium is formed [1]. Then, the stem internodes elongate rapidly, a process called bolting and inflorescence expansion, and the flowers bloom for a few days. Because lettuce is a crop grown for its foliage, early bolting is an unwelcome characteristic. Prolonging the nutritional period is conducive to leaf biomass accumulation and quality maintenance [2]. Very early varieties start bolting and flowering after planting for 7 weeks in a greenhouse, while very late varieties may bloom after planting for 4 months. Premature bolting can lead to partial or total yield loss, especially in early autumn planting in hot production areas. Therefore, the genetic basis of bolting-related regulation has been the focus of many studies for many years [3].

Inhibition of early bolting can help stabilize the yield [4,5]. Bolting and flowering are promoted to shorten the breeding cycle and speed up the breeding process. The bolting of vegetables is regulated by various endogenous and exogenous hormones, such as auxin, gibberellin, brassinolide, and ethylene [6], promoting bolting, while paclobutrazol [7] can inhibit bolting. Gibberellin (GA_3_) plays an important role in the bolting of vegetables. GA_3_ can break dormancy and promote bud development [8]. It was found that GA_3_ strongly promoted the bolting of Chinese cabbage, and as the GA_3_ concentration increased, this effect was enhanced [9]. As the first plant hormone discovered and isolated, IAA has been extensively studied. In the same plant, different concentrations of auxin usually have different effects. There is synergism and antagonism between plant hormones. Luckwill first proposed the hypothesis of “hormone balance” [10], in which this equilibrium state controls the metabolism of various substances, thus regulating the growth and development of plants [11].

Leaf lettuce (*Lactuca sativa* L.) is a vegetable in the Compositae family. Because of its crisp taste, rich nutrients, low carbohydrate content, and high level of dietary fiber [12], this vegetable has become increasingly popular in recent years. However, there have been few studies on the relationship between exogenous hormones and bolting of leaf lettuce, and most treatments in previous studies used single hormones. Few studies have explored the effect of spraying exogenous hormones on the bolting of leaf lettuce. The application of exogenous hormones can regulate the breeding cycle and provide better strategies for breeding. Studies have shown that gibberellin can partially or completely replace low-temperature vernalization to induce bolting and flowering ahead of time [13,14]; as the earliest discovered and isolated plant hormone, auxin plays a role in flower initiation [15,16]. Therefore, it is important to further clarify the relationship between exogenous auxin and gibberellin and their synergistic effects on the bolting of leaf lettuce.

## 1 Materials and methods

### 1.1 Experimental details and treatments

#### 1.1.1 Experimental details

The variety of leaf lettuce used in these experiments is the germplasm material GB-31 preserved in the laboratory. The experiments were carried out in a multispan greenhouse on the eastern side of the Beijing University of Agriculture from January to June 2018. The initial conditions included light/dark (14 h/10 h) and day temperature/night temperature (20 ± 2°C/13 ± 2°C). The relative humidity was 50–70%. When the plants grew to six leaves, the culture conditions were as follows: day temperature/night temperature (20/13°C); light/dark (14 h/10 h); relative humidity: 60%; and spraying.

The seeds of GB-31 were sown in a 5 × 10 hole plug tray. When the seedlings had four leaves and one heart, they were transplanted to a nutrient bowl with a diameter of 15 cm. The ratio of the matrix used was peat:vermiculite:perlite = 2:1:1. When the lettuce had six leaves, plants at the same growth stage and good growth conditions were selected for the experimental treatments.

#### 1.1.2 Treatments

There were four treatments in the experiments: 40 mg/L IAA, 25 mg/L GA_3_, 40 mg/L IAA + 25 mg/L GA_3,_ and water spray that was used as a control (CK). The stem length was measured every 3 days after treatment with 10 replicates per treatment. After the IAA treatment, the samples were observed at 0, 2, 4, 6, 8, 12, 18, and 24 days after spraying. After treatment with GA_3_ and IAA + GA_3_, the samples were observed at 0, 2, 4, 6, 8, 12, and 16 days after spraying. The control group was sampled and observed at 0, 2, 4, 6, 8, 12, 16, 18, and 24 days after spraying. The sampling site was the stem, selected from the upper part within 1 cm below the shoot apex. For endogenous hormone determination, three plants were randomly selected from each treatment group, and three replicates were used for each treatment.

### 1.2 Determination methods

#### 1.2.1 Physiological Index

After adding exogenous hormones, 10 plants were randomly selected from each treatment group every 3 days, and the stem length was measured and recorded with a measuring tape. The stem length was measured from the cotyledon to the shoot apex. The routine paraffin sectioning method was used for cytological observation, and the safranin-fast, green-dyeing method was used. The slice thickness was 10 μm, and a fluorescence microscope was used to observe and take photos at a scale of 200 μm. Endogenous hormones were determined by enzyme-linked immunosorbent assay (ELISA). The ELISA kit was provided by China Agricultural University. The tested hormones were gibberellin (GA), auxin (IAA), abscisic acid (ABA), and brassinolide (BR).

#### 1.2.2 Quantitative fluorescent PCR

To detect the expression of gibberellin biosynthesis- and transduction-related enzymes, we selected related genes for fluorescence quantitative PCR by reading the literature. 18 S ribosomal RNA was used as an internal reference gene, and a reaction system of 10 μL was as follows (Table 1).

**Table 1:** System for the quantitative determination of gene expression by real-time fluorescence

Material	Volume (μL)
cDNA	1
Primer 1	1
Primer 2	1
SYBR Green	5
DEPC-treated ddH_2_O	2
Total Volume	10

The cDNA obtained by reverse transcription was subjected to quantitative fluorescent PCR with a 5-fold gradient dilution (1, 1/10, 1/100, 1/1,000, and 1/10,000). The reaction procedure was as follows: predenaturation at 95°C for 3 min; denaturation at 95°C for 20 s, annealing at 53°C for 20 s, and extension at 72°C for 20 s (40 cycles); finally, 65–95°C, with every rise of 0.5°C for 30 s; and the fluorescence value was detected and the dissolution curve was drawn. The reaction procedure of the internal reference gene was the same as that of the target gene, and each sample was assayed three times. The 2^−△△Ct^ method was used for calculations [17].

#### 1.2.3 Statistical analysis

Excel 2016 was used for data processing, the test data are expressed as the mean ± standard deviation, and the standard deviation was calculated by *t*-tests. SPSS 20.0 software was used to analyze the significance of differences, and OriginPro 9.0 software was used for mapping.

## 2 Results

### 2.1 Effects of exogenous auxin and gibberellin on lettuce bolting

**Figure 1:**
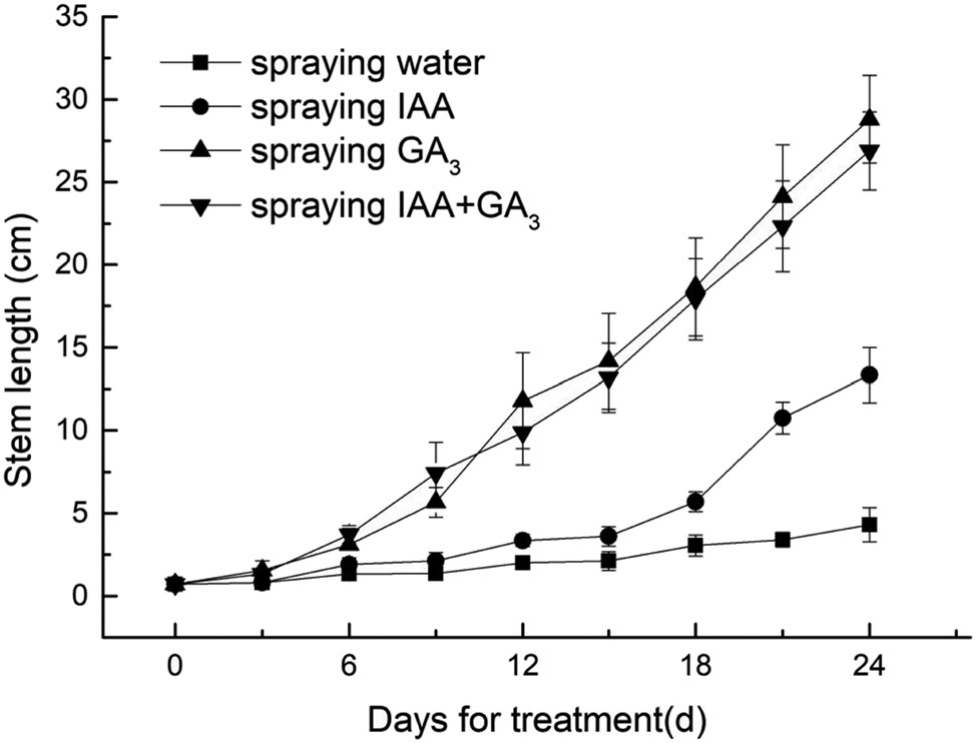
Stem length changes after treatment with GA_3_ or IAA.

Spraying the plants with gibberellin alone, auxin alone, or a combination of both could promote the stem elongation of lettuce, but the effect was different (Figure 1). Over time, the stem length of leaf lettuce was significantly different. After applying GA_3_ and a combination of GA_3_ and auxin, the stem length of leaf lettuce grew faster. On the third day, the stems treated with exogenous gibberellin or gibberellin and auxin showed obvious elongation, and the stem lengths reached 1.55 and 1.33 cm, respectively, which were significantly different from that of the control group. On the 6th day, there was a significant difference between the two groups, and the treatment with a combination of auxin and gibberellin resulted in longer stems than the treatment with GA_3_ alone. On the 12th day, the stem length of the group treated with GA_3_ alone was longer than that of the group treated with auxin and GA_3_. The application of auxin alone also promoted the stem elongation of lettuce, but the effect was slow. On the sixth day, the stem length of the group treated with auxin alone was 1.9 cm, which was 1.43 times longer than that of the control group. This difference was significant from the 15th day. In the control group, the stem length changed slowly. On Day 0, the stem length was 0.7 cm. On the 24th day, the stem length was only 4.3 cm, and the rate of stem elongation was much slower than that of the hormone treatment groups.

### 2.2 Microscopic observation of the shoot tip of lettuce treated with exogenous auxin and gibberellin

**Figure 2:**
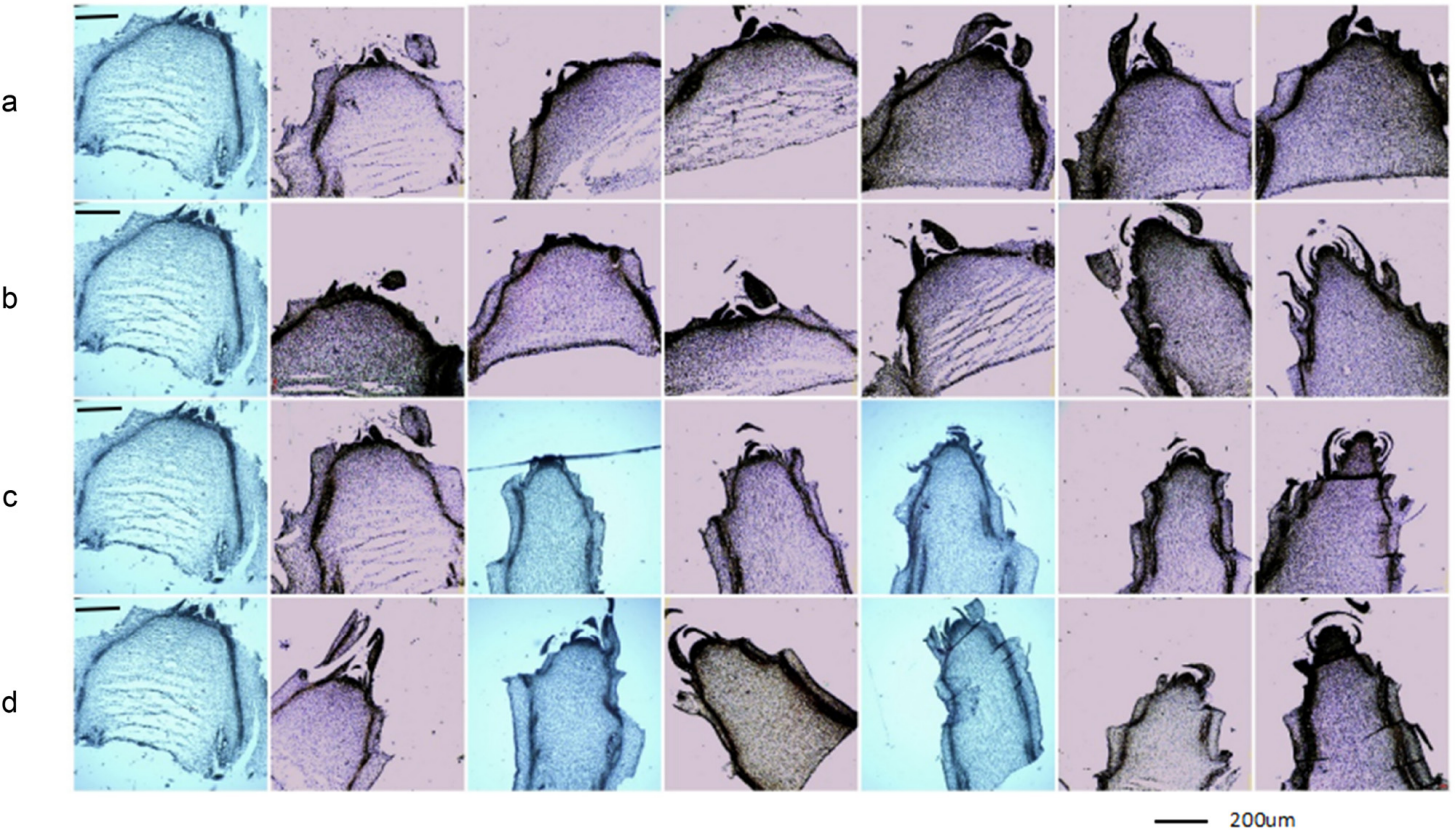
Comparison of the flower bud differentiation of lettuce after treatment with GA_3_ or IAA. **Note:** A, B, C, and D represent the control group, exogenous IAA treatment group, exogenous GA_3_ treatment group, and combined IAA and GA_3_ treatment group, respectively. (a and b) Represent flower bud differentiation at 0, 2, 6, 8, 12, 16, and 24 days from left to right. (c and d) Represent flower bud differentiation at 0, 2, 4, 6, 8, 12, and 16 from left to right.

As shown in Figure 2, on the fourth day, the shoot tip became thinner, the growth point changed from conical to flat. The flower bud began to differentiate, and the growth point entered the stage of the hypertrophic growth. On the 6th day, a point-like protuberance was formed around the growth point, multiple involucres and primordia were differentiated and bent inward, and the scales of involucre entered the initial stage of differentiation. On the 12th day, the primordium of the involucre elongated upward and completely wrapped the growth point and entered the final stage of involucre differentiation. On the 16th day, irregular granular bulges were formed around the growth point, and floret primordium differentiation occurred. In the group treated with auxin alone, the flower bud differentiation rate was slow. On the 8th day, this group entered the hypertrophic growth stage. On the 18th day, a bulge was formed around the growth point, and the involucre primordium was formed. On the 24th day, the involucre primordium extended upward to wrap the growth point and entered the final stage of involucre differentiation. However, in the control group, on the 18th day, flower bud differentiation appeared, and the hypertrophic growth stage occurred.

### 2.3 Changes in endogenous hormonal contents in lettuce stem after exogenous auxin and gibberellin application

#### 2.3.1 Changes in the GA_3_ content in lettuce stems after exogenous auxin and gibberellin treatment

The GA_3_ content in the stem of leaf lettuce was 24.1 ng/g FW on Day 0. With the passage of treatment time, the content of GA_3_ in the stem of leaf lettuce decreased. The content of GA_3_ in the stems of the treatment group was slightly higher than that of the control group; the content of GA3 following exogenous spraying treatment was higher than that following the other treatments.

#### 2.3.2 Changes in IAA content in lettuce stems treated with exogenous auxin and gibberellin

**Figure 3:**
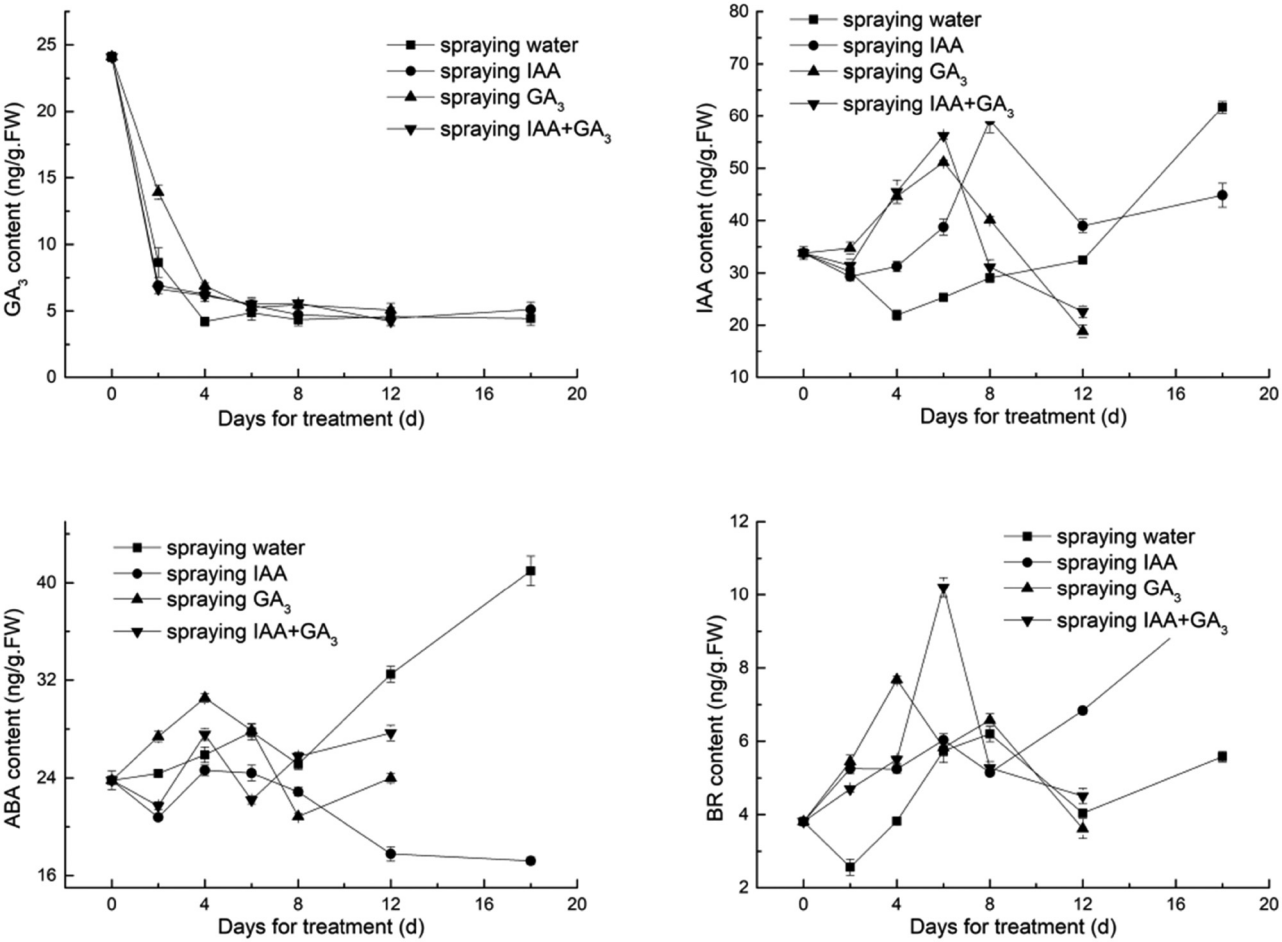
Changes in endogenous hormones in the stems of lettuce after treatment with GA_3_ or IAA.

As shown in Figure 3, with the extension of treatment time, the IAA content in the stems of plants treated with hormones showed a similar pattern: the level first increased, followed by a decrease and another increase. The content of IAA reached a peak on the 6th day after treatment with exogenous GA_3_ and combined IAA and GA_3_ and was significantly different from that of the control group. At this time, the endogenous IAA concentration reached 51.17 and 56.20 ng/g FW, respectively, and then decreased; the IAA concentration in the stems of the group treated with auxin alone peaked on the 8th day and then decreased, and the IAA content in the early treatment group was higher; on the 12th day, the content of IAA in the control group was higher than that of the groups treated with exogenous hormones.

#### 2.3.3 Changes in the ABA content in lettuce stems treated with exogenous auxin and gibberellin

As shown in Figure 3, the ABA content in the stems of leaf lettuce treated with IAA alone and combined IAA and GA_3_ was lower than that in the control group, and the ABA content in the stem decreased and was lower than that at 0 days. The content of endogenous ABA in stems increased after exogenous GA_3_ application. On the fourth day, the ABA content in the stems of the groups treated with GA_3_ alone or combined IAA and GA_3_ increased briefly and then decreased. After treatment with exogenous IAA, the ABA concentration decreased slowly and began to decline on the 8th day. From the 6th day, the ABA content in the control group was gradually higher than that in the groups treated with exogenous hormones.

#### 2.3.4 Changes in BR content in lettuce stems treated with exogenous auxin and gibberellin

With the prolongation of treatment time, the BR content in the stems of the treatment group was higher than that of the control group in the early stage after the application of GA_3_ and combined IAA and GA_3_, and the highest values were found on day 4 and day 6, respectively, followed by a decrease. After treatment with IAA alone, the BR content in the stem increased slowly in the early stage and was lower than that after treatment with exogenous GA_3_ and the mixture of IAA and GA_3_; overall, the content of BR continued to increase and was significantly higher than that of other treatment groups after 12 days.

### 2.4 Effect of exogenous auxin and gibberellin on gene expression related to hormone signal transduction

**Figure 4:**
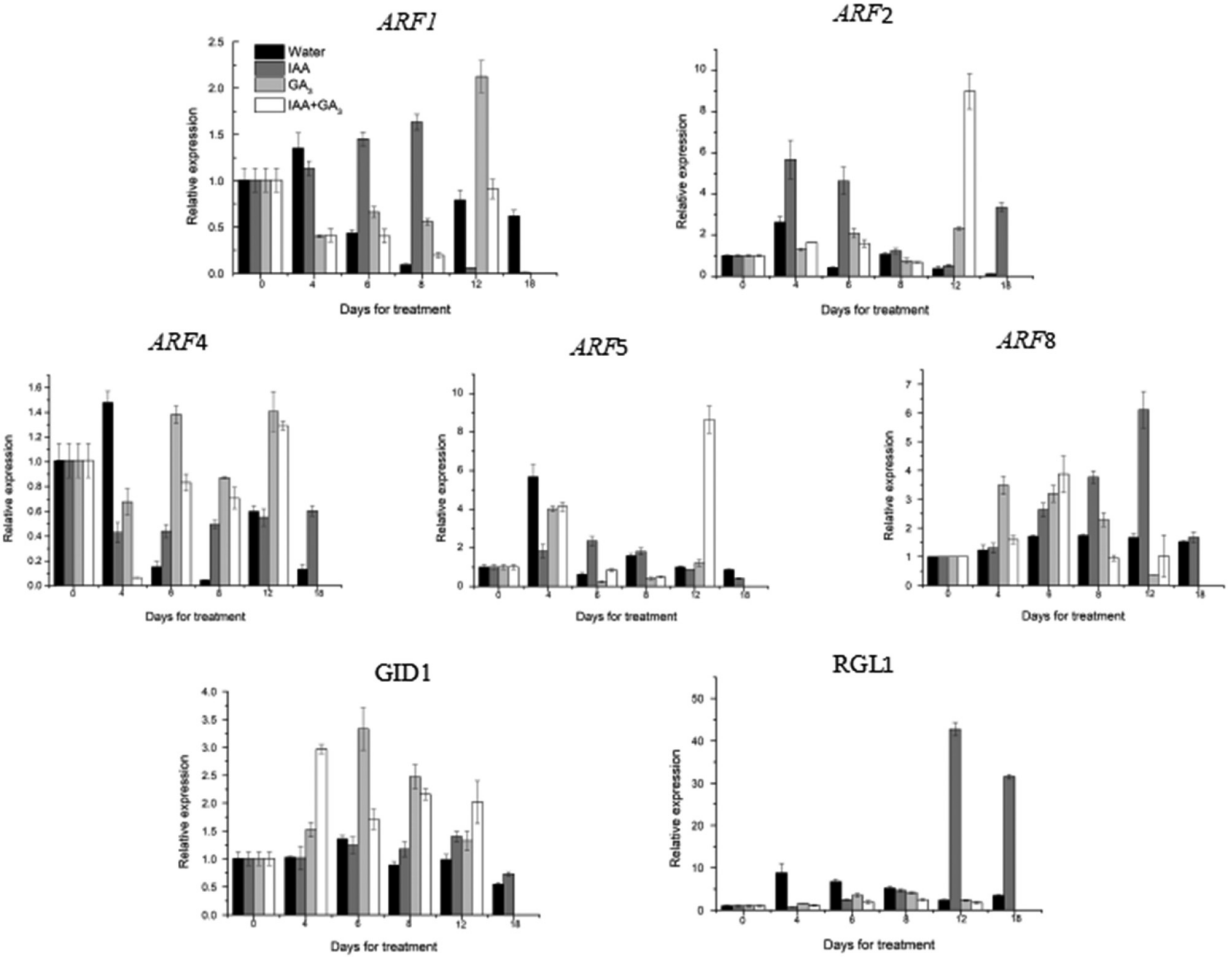
Analysis of the mRNA expression levels of auxin and gibberellin signal transduction-related genes during leaflet lettuce development after treatment with GA_3_ or IAA. Note：**indicates a significant difference at *P* ≤ 0.01, and *indicates a significant difference at *P* ≤ 0.05.111.

Based on previous studies in our laboratory, the *ARF1*, *ARF2*, *ARF4*, *ARF5*, and *ARF8* genes that may be related to bolting and flowering were selected to measure their relative expression in different treatments. On the 4th day, the relative expression of the *ARF1* gene in the groups treated with exogenous IAA, GA_3_, and combined IAA and GA_3_ was lower than that of the control group, while the relative expression of the *ARF1* gene was downregulated by GA_3_ alone or by a combination of IAA and GA_3_. On the 8th day, the relative expression of *ARF1* in the groups treated with GA_3_ and combined IAA and GA_3_ was higher than that of the control group. On the 12th day, the relative expression of *ARF1* decreased significantly in the group treated with IAA alone but increased in the group treated with GA_3_ and combined IAA and GA_3_. On the 18th day, the relative expression of *ARF1* in IAA-treated plants continued to decrease and was only 0.016 times that in the control. For the *ARF2* gene, the relative expression level in the groups treated with GA_3_ and combined IAA and GA_3_ was low in the early stage and increased rapidly on the 12th day, reaching a level that was 6.2 times and 24.2 times that of the control group, respectively. The expression level of IAA was higher on the 4th and 18th days. The relative expression of the *ARF4* gene decreased on the 4th day, increased from the 6th day and was maintained at a high level. On the 4th day, the relative expression of the *ARF5* gene in the treatment group was lower than that in the control group and then remained at a low level. On the 12th day, the relative expression of the *ARF5* gene in the group treated with combined IAA and GA_3_ was significantly upregulated compared with the control. The relative expression of the *ARF8* gene was upregulated on the 4th and 6th days, decreased on the 8th day, and was higher than that in the control group on the 12th day. After administration of IAA, the *ARF8* gene was significantly upregulated on the 8th and 12th days and was downregulated on the 18th day. For the *GID1* gene, the GA_3_ and combined IAA and GA_3_ treatments increased the expression from the 4th day and decreased it from the 8th day, but the relative expression remained at a relatively higher level. The relative expression level of *GID1* after IAA treatment was almost the same as that of the control group on the fourth day, increased slightly on the eighth day, and remained at a higher level than that of the control group. The expression level of the *RGL1* gene increased from the 4th day after the application of GA_3_ and combined IAA and GA_3_, peaked on the 8th day, and then decreased. The relative expression of IAA on the 12th and 18th days was 18.2 and 9.4 times higher than that of the control group, respectively, and the difference was significant (Figure 4).

## 3 Discussion

In the process of lettuce cultivation, lettuce shortening, stem elongation, and even bolting will affect the “leaf” harvest of lettuce, resulting in the decline of lettuce yield [18]. “GB-31,” as an easy bolting variety, is in line with the research direction of this experiment, so it is selected as the experimental material. Previous studies have shown that the concentrations of GA3 and auxin are positively correlated with bolting rate and bolting height. Therefore, it is speculated that spraying auxin and gibberellin will have a certain effect on lettuce bolting. *ARF* is an auxin-responsive factor and GID1 is a gibberellin receptor. The expression of the *ARF* gene and GID1 is helpful to further explore the bolting mechanism of leaf lettuce between auxin and gibberellin [19].

Gibberellin is an important plant hormone, which participates in many processes of regulating plant physiology [20]. One of the more prominent characteristics is to promote shoot tip elongation and plant height. Lettuce bolting is stimulated by environmental changes such as temperature and sunshine length. With the differentiation of flower buds, the stem begins to elongate rapidly and the plant becomes higher, which has a great relationship with this experimental treatment. In addition, through paraffin sectioning and whole plant photograph observation, it was found that spraying auxin promoted the lateral width of the stem. At the same stage of flower bud differentiation, the stems treated with auxin were thicker compared with other treatments, and the stems treated with combined gibberellin and auxin were thicker than those treated with gibberellin alone. Liu et al. found that after bolting lettuce with heat-treated leaves, the levels of GA3 and GA4 and the content of indole acetic acid (IAA) in stems increased sharply and remained stable. Further study found that the effect of GA3 and IAA mixed spraying was better than that of single spraying, and the bolting phenomenon occurred in 4 days. This finding is similar to the conclusion that GA3 and IAA combined application on flue-cured tobacco has a better effect on improving quality than a single application [11]. At the same time, this experiment found that the bolting time of IAA alone was relatively late, but it can promote the increase of endogenous GA3 content in the stem. It is speculated that the effect of mixed spraying is more obvious, which may be related to IAA promoting the increase of GA3 content and further promoting the elongation and growth of the stem.

Different hormones have different regulatory effects on plant bolting, some play a direct role, some play an indirect role, and then affect lettuce bolting by affecting the expression of other hormones [21,22]. As the earliest discovered plant hormone, auxin plays an important role in the whole process of plant growth and development. It is not only involved in regulating cell elongation, division and differentiation, and apical dominance but also involved in signal transduction and flower development. Previous studies have suggested that a low concentration of IAA promoted bolting, while a high concentration of IAA was not conducive to bolting [23,24]. In recent years, it has been found that a low concentration of IAA at an early stage promotes bolting and flowering, but a higher IAA concentration is required at the bolting stage [25]. In this study, although different treatments resulted in different times of bolting, the trend of change in the endogenous IAA content of the stems was similar, with higher concentrations at the beginning of bolting, lower concentrations in the early stage of bolting, and a gradual decrease after bolting. which is basically consistent with the previous research results, which further proves that IAA is involved in the bolting of lettuce.

*ARF* protein family is a key protein in response to auxin signal and plays an important role in the auxin signal transduction pathway. There are many members of the *ARF* family, and there are great functional differences among different *ARF* proteins. So far, 23 at *ARF* genes have been found in Arabidopsis. The *ARF1* gene plays an important role in plant growth and vesicle transport [26], maintenance of some organelles [27,28], and antiviral defense. After exogenous IAA application, *ARF1* gene expression in the stem remained at a high level in the early stage; treatment with GA_3_ or a combination of IAA and GA_3_ led to increases on the 8th day; however, the relative expression of the *ARF1* gene in the IAA treatment group was much higher than that in the other two hormone treatment groups. It was speculated that exogenous IAA promoted *ARF1* gene expression and that GA_3_ inhibited *ARF1* gene expression. Yang Xiao [29] and others found that the *ARF2* gene was expressed at high levels in tomato flower buds. In this study, the relative expression levels in the groups with the GA_3_ and combined IAA and GA_3_ treatments were lower in the early stage and increased rapidly on the 12th day, which was the final stage of involucre scale differentiation. The expression level in the group treated with IAA alone was higher on the 4th and 18th days. The expression of the *ARF2* gene was upregulated on the 18th day after IAA treatment, which may have been due to preparation for floret primordium differentiation. In the *Arabidopsis thaliana*, the *ARF4* gene is involved in flower development [30]. In this study, we found that the relative expression of the *ARF4* gene was lower after the application of GA_3_ alone, IAA and GA_3_ combined, and IAA on the 8th day; that is, the expression of the *ARF4* gene decreased at the beginning of flower bud differentiation. However, it increased gradually after bolting, which indicated that the *ARF4* gene may play a role in promoting the late bolting stage [31]. In this study, when GA_3_ and IAA and GA_3_ were applied, the relative expression of the *ARF8* gene was lower on the 4th to 8th days and the 12th day. After the application of exogenous IAA, the expression level was upregulated from Day 8 to Day 12 and downregulated on Day 18. The results showed that the *ARF8* gene promoted bolting [32].

*GID1* is a soluble protein-encoding gibberellin. It combines with GA to recognize DELLA in the nucleus, resulting in polyubiquitination of the DELLA protein, degradation of DELLA protein, and release of gene expression inhibited by DELLA [13]. In this study, compared with that of the control group, the expression of the *GID1* gene was higher in the group sprayed with IAA, GA_3_ alone, and the mixture of IAA and GA_3_ at the beginning of bolting, indicating that the *GID1* gene may promote bolting. After the three treatments, the expression of *RGL1* increased gradually after bolting and then decreased slowly, with the highest concentration at the stage of bulb scale differentiation. It is speculated that the possible mechanism is that the degradation of DELLA makes a large number of gibberellin express, to induce the rapid growth of lettuce stems and bolting. The expression of *RGL1* was lower in the early stage of bolting, and the relative expression of *RGL1* was higher after bolting. These results indicated that *RGL1* played an important role in flower development. In research on *Arabidopsis thaliana*, it was also found that RGL1 plays an important role in regulating flower bud differentiation and flower development [33,34].

In conclusion, based on the rapid elongation of stems and microscopic observation of flower bud differentiation, the combination of gibberellin and auxin has a synergistic effect. A combination of exogenous applications of gibberellin and auxin can promote leaf lettuce bolting. *ARF8* and *GID1* are involved in the bolting leaf lettuce. *ARF1*, *ARF2*, *ARF4,* and *RGL1* may participate in flower development, but the relationship between the *ARF5* gene and bolting and flowering is not clear.
